# 

**Published:** 1889-05

**Authors:** 


The Thirteenth Annual Meeting of the New Hampshire Dental Society will be held at Concord, June 18 and 19, 1889. All members of the profession invited to attend. Reduced railroad fares. The Board of Censors will meet any candidate for license Monday evening, June 17th, at the office of the Secretary.

EDWARD B. DAVIS, *Secretary.*

---

### CHICAGO DENTAL SOCIETY.

At the annual meeting of the Chicago Dental Society, held on Tuesday evening, April 2, 1889, the following named were elected officers for the ensuing year: P. J. Kester, President; D. M. Cattell, First Vice-President; W. J. Martin, Second Vice-President; A. E. Baldwin, Secretary; Louis Ottofy, Corresponding Secretary; E. D. Swain, Treasurer; A. W. Harian, Librarian; Member of the Executive Committee, J. Austin Dunn; Board of Censors, F. H. Gardiner, C. F. Hartt and L. L. Davis. Delegates to the International Dental Congress at Paris, France, September 1 to 8, 1889, were appointed, as follows: A. W. Harlan (Secretary), J. N. Crouse, T. W. Brophy, J. A. Swasey, P. J. Kester, W. W. Allport, A. E. Baldwin, Louis Ottofy, L. L. Davis, J. W. Wassall, and W. B. Ames.

A report from Dr. Crouse showed the Dental Protective Association of the United States to be flourishing, and dentists throughout the United States are requested to become members of this Association.

LOUIS OTTOFY,

*Corresponding Secretary.*

SUBSCRIBE FOR THE

### International Dental Journal

**The Organ of the Profession,**

PUBLISHED BY THE

### INTERNATIONAL DENTAL PUBLICATION COMPANY:

**LOUIS JACK,** *President,* Philadelphia.
**BENJAMIN LORD,** *Vice President,* New York.
**C. N. PEIRCE,** *Treasurer,* Philadelphia.
**GEO. S. ALLAN,** *Secretary,* 51 W. 37th St., New York

---

**W. XAVIER SUDDUTH, M D., D.D.S., F.R.M.S.**

Editor and Business Manager,

1215 Filbert Street, Philadelphia, Pa.

---

### FOREIGN CORRESPONDENTS:

Dr. ARKÖVY, Budapesth, Hungary.
W. GEO. BEERS, D.D.S., Ed. Dominion Dental Journal, Montreal.
L. C. BRYAN, D. D. S., Basel.
ALFRED BURNE, D.D.S., Sydney, New South Wales.
F. BUSCH, M.D., Berlin.
GEORGE CUNNINGHAM, Cambridge, Eng.
I. B. DAVENPORT, D.D.S., Paris.
PAUL DUBOIS, Ed. L'Odontologie, Paris.
WILLIAM DUNN, Jr., L.D.S., Florence.
A. H. EDWARDS, D.D.S., Madrid.
W. ST. GEORGE ELLIOTT, M.D., D.D.S., Lon.
ALPHONSE FEUCHEL, Zahnarzt, St. Petersburg.
C. R. FLETCHER, D.D.S., Sao Paulo, Brazil.
WALTER HARRISON, L.D.S., D.M.D., Brighton, Eng.
ROBERT S. IVY, D.D.S., Shanghai, China.
N. S. JENKINS, D.D.S., Dresden.
F. KLAPPROTH, Zahnarzt, St. Petersburg.
L. KOLBE, " " "
Dr. GEO. P. E. KUHN, Paris.
W. BOWMAN MACLEOD, L.D.S., Edinburgh
W. D. MILLER, Ph. D., M.D., D.D.S., Berlin.
WILLIAM SACHS, D.D.S., Breslau.
J. G. VAN MARTER, B.A., D.D.S., Rome.
J. M. WHITNEY, M.D., D.D.S., Honnolula.
LUDWIG WARNEKROS, Zahnarzt, Berlin.
LUDW. ADOLPH WEIL, M.D., Munich.
J. COWAN WOODBURN, M.D., Glasgow.

---

### STOCKHOLDERS:

Geo. S. Allan,
R. R. Andrews,
H. A. Baker,
W. C. Barrett,
A. P. Beale,
S. T. Beale, Jr.
C. S. Beck,
G. V. Black,
E. A. Bogue,
C. F. W. Bodecker,
W. G. A. Bonwill,
C. A. Brackett,
Edward C. Briggs,
Thos. Bradley,
Albert H. Brockway,
A. E. Brown,
Wm. Carr,
Geo. H. Chance,
G. F. Cheney,
Dwight M. Clapp,
John D. Codman,
Chas. D. Cook,
J. N. Crouse,
Edw. T. Darby.
D. M. Davidson,
Wm. H. Dwinelin.
Chas. J. Essig,
J. N. Farrar,
T. Fillebrown,
W. B. Finney,
T. A. Fletcher,
M. W. Foster,
Chas. E. Francis,
W. F. Fundenberg,
W. H. Fundenberg,
E. C. Fundenberg,
A. H. Gilson.
E. S. Gaylord,
J. P. Geran,
S. H. Guilford,
John I. Hart,
O. E. Hill,
J. F. P. Hodson,
J. Morgan Howe,
Robert Huey.
A. O. Hunt,
Louis Jack,
C. R. Jefferis,
C. A. Kingsbury,
Edw. C Kirk,
C. R. E. Koch,
W. T. La Roche,
W. K. Leech,
F. A. Levy,
Wilbur F. Litch,
J. Bond Littig,
Benjamin Lord,
Geo. W. Lovejoy (Canada),
John S. Marshall,
James McManus,
Charles McManus,
E. V. McLeod,
H. J. McKellops,
Dan'l Neal McQuillan,
Horatio C. Meriam,
W. D. Miller, (Berlin),
W. E. Page,
S. B. Palmer,
C. B. Parker,
D. D. Peabody,
S. G. Perry,
C. N. Peirce,
Chas. E. Pike.
W. A. Phreaner,
W. E. Pinkham,
W. H. Potter,
H. C. Register,
Z. T. Sailer,
L. D. Shepard,
Eugene H. Smith,
B. Holly Smith,
H. A. Smith,
S. G Stevens,
W. X. Sudduth,
Ambler Tees,
J. D. Thomas,
James Truman,
Wm. H. Trueman,
F. F. Van Woert,
W. W. Walker,
I. F. Wardwell,
C. S Wardwell,
G. W. Warren,
T. S. Waters,
Geo. W. Weld.
Julius G. W. Werner,
Jacob L. Williams.
R. B. Winder,
C. A. Woodward,
J. A. Woodward,
W. J. Younger.

